# Reply to Arnold et al.: More than words? Defining best practices in assessing surrogate understanding

**DOI:** 10.1093/atsscholar/aapag007

**Published:** 2026-01-30

**Authors:** Kristen E Pecanac, Blair P Golden

**Affiliations:** University of Wisconsin–Madison School of Nursing, Madison, WI, United States; Department of Medicine, University of Wisconsin–Madison School of Medicine and Public Health, Madison, WI, United States

From the Authors:

We appreciate the letter by Arnold and colleagues in contributing to this important discussion of defining best practices in assessing surrogate understanding. The authors contend that “competence to lead these conversations likely hinges less on individual words and more on a clinician’s ability to consistently exhibit a core set of skills in every clinical encounter.” We absolutely agree that a clinician’s ability to exhibit a core set of skills in every clinical encounter is essential to improving communication. What we are adding to this conversation is that we (and others^1^) contend that analyzing word choice and phrasing is essential to inform the core set of skills necessary for surrogate decision-making ­conversations. 

Experts in conversation analysis have described conversations as a series of sequences that complete activities.^2^ Questions asked during a conversation help to move the conversation forward to complete these activities, such as shared understanding of the patient’s clinical situation. Arnold and colleagues describe 2 “tasks” of assessing surrogate understanding: (1) elicit understanding of the current medical situation, and (2) elicit understanding of what the current medical situation means for the future. Both tasks are essential. However, to get to the second task, you need to complete the first task. Our analysis was meant to decipher how best to elicit surrogate understanding of the current medical situation (task #1) in order to move the conversation forward to elicit understanding of what the current medical situation means for the future (task #2). Our analysis shows that asking what surrogates “understood” or “what is going on” helps to move the conversation forward toward task #2, or the “big picture” implications of the patient’s situation.^3^ Asking what surrogates “heard,” what they “know,” or what “has happened” merely led to the retelling of events and assessments, responses that do not help clinicians to know whether surrogates understand the implications of what they are retelling.^3^

The notion that word choice or phrasing can have major implications for conversations is not new. Asking “Do you have any questions or concerns?” is more likely to lead a “no” response than asking about “what concerns” or “some other concerns.”^4^ Clinicians who begin treatment recommendations for a child with a viral infection stating that they are not offering antibiotics are more likely to encounter parent resistance than those that begin with an appropriate treatment.^5^ In surrogate decision making, asking “What would the patient want?” is more likely to lead to a surrogate response than stating “We need to know what the patient would want,” which surrogates respond to as a statement, not a question (“mm hmm,” “okay”).^6^ Knowing how people typically respond to different statements or questions is critical for achieving conversational activities.

Overall, showing the implications of choosing one word or phrase over another can add value to current communication trainings. This type of analysis is not meant to replace or minimize the great work that has already been done but rather to complement the work and provide guidance on a more granular level to keep the conversation moving forward to complete the necessary activities. Achieving shared understanding is a difficult yet essential task for shared decision making. We need all our tools to make this possible.

## Supplementary Material

aapag007_Supplementary_Data
